# The Prevalence of Fibromyalgia in Other Chronic Pain Conditions

**DOI:** 10.1155/2012/584573

**Published:** 2011-11-17

**Authors:** Muhammad B. Yunus

**Affiliations:** Section of Rheumatology, Department of Medicine, University of Illinois College of Medicine at Peoria, One Illini Drive, Peoria, IL 61605, USA

## Abstract

Central sensitivity syndromes (CSS) include fibromyalgia syndrome (FMS), irritable bowel syndrome, temporomandibular disorder, restless legs syndrome, chronic fatigue syndrome, and other similar chronic painful conditions that are based on central sensitization (CS). CSS are mutually associated. In this paper, prevalence of FMS among other members of CSS has been described. An important recent recognition is an increased prevalence of FMS in other chronic pain conditions with structural pathology, for example, rheumatoid arthritis, systemic lupus, ankylosing spondylitis, osteoarthritis, diabetes mellitus, and inflammatory bowel disease. Diagnosis and proper management of FMS among these diseases are of crucial importance so that unwarranted use of such medications as corticosteroids can be avoided, since FMS often occurs when RA or SLE is relatively mild.

## 1. Introduction: Historical Overview and the Importance of Nomenclatures

The fact that fibromyalgia syndrome (FMS) is associated with several similar conditions without structural pathology was first reported in a controlled study in 1981 [1], following which a conceptual model was proposed with a Venn diagram, showing the mutual overlaps in these syndromes [2]. Since then, a large number of studies have confirmed these associations, compared with both healthy controls as well as diseases with structural (the so-called “organic”) pathology (DWSP) [3, 4]. (Although there are structural changes, e.g., decreased hippocampus and gray matter volume in some CSS conditions, these changes seem to result from prolonged central sensitization (CS) and will be discussed at the end.) These overlapping conditions are collectively known as central sensitivity syndromes (CSS) [5–8], since CS is the common binding glue between them. The term CSS was first coined in 2000 [5] and has been reviewed [5–8]. We [6] and others [9] have described mutual associations among the CSS conditions.

In contrast to association of FMS with other CSS members, association of FMS with DWSP was reported somewhat later [10] and more frequently only in recent years. Wolfe and Cathey were the first to recognize the association of FMS with rheumatoid arthritis (RA) [10]. Although they were called “secondary fibrositis,” these were cases of concomitant FMS with typical symptoms and multiple tender points (TP) in association with RA. The diagnosis of FMS in the pre-1990 ACR criteria era was made on the characteristic symptoms of FMS as well as many tender points [10]. 

What's in a name? Asked Shakespeare rhetorically in Romeo and Juliet. My own answer is “A lot.” A name should be meaningful, tell the gist of the topic, and must not distort the underlying truth, recognizing that scientific truth is not carved in rock and does change over time. In this discourse, I shall use the terms syndrome, illness, condition, and disease synonymously. Further, since the term fibromyalgia implies nothing more than pain as has been discussed by Fitzcharles and Yunus in this issue of the journal, I prefer the term “fibromyalgia syndrome” since FMS is so much more than pain with a large number of other distressing symptoms.

Elsewhere, I have argued that differentiation between illness and disease is artificial and contrary to patient interest and hampers proper management of CSS conditions, since anything that is not currently viewed as a disease (i.e., does not have structural pathology, e.g., inflammation, degeneration, or neoplasm) is viewed as predominantly or exclusively psychological and benign and is not taken seriously by the health care providers, accentuating the suffering of the patients [7]. In an irresponsible way, this untruthful “dogma” is passed down from the professor or the attending to the students, and the victims are our patients. 

I have also rationalized that the use of the term “organic” for DWSP is irrational, since organs are involved in both CSS and DWSP—functional changes in the former and structural in the latter [7]. In the same context, the use of the nosology “functional” for CSS is antithetical, since the neurochemical-endocrine status of organs involved in CSS is dysfunctional! 

Such nomenclatures as “medically unexplained symptoms” or MUS and “somatization disorder” (SD) are equally fallacious and detrimental to scientific progress, and statements of bias. Such a bias impedes empathetic and proper patient care. By DSM IV-TR definition, in SD “laboratory tests are remarkable for the absence of findings to support subjective symptoms.” This is obviously not true of CSS diseases as has been adequately discussed [7]. CSS are medical conditions based on objective pathology of neurochemistry and neuroimaging that explain many symptoms of CSS diseases [7]. So, terms like MUS seem to sprout in a fertile mind of bias. The problem is that MUS represents illness and illness is a second class citizen in the land of medicine. 

## 2. Materials and Methods: Process of Locating Articles for This Review and the Author's Own Views

Several sources of literature search were employed: PubMed, Science Citation Index—ISI Web of Knowledge and CINAHL Plus. A large number of key words were used, including fibromyalgia, fibromyalgia syndrome, functional syndromes, somatic syndromes, medically unexplained symptoms, CS, central sensitivity syndromes, overlapping conditions, chronic pain, individual members of central sensitivity syndromes (e.g., irritable bowel syndrome, headaches, migraine, temporomandibular disorder, myofascial pain syndrome, restless legs syndrome, vulvodynia, and interstitial cystitis), chronic back pain, chronic neck pain, chronic pelvic pain, and chronic disease, as well as a number of chronic pain conditions with structural pathology (e.g., RA, systemic lupus erythematosus, osteoarthritis, ankylosing spondylitis, diabetes mellitus, ulcerative colitis, Crohn's disease, endometriosis, carpel tunnel syndrome, and neuritis). Additionally, all references of a given article were searched for an additional source. Finally, this paper adds my own ideas.

## 3. FMS in CSS Diseases: Associations Are Bidirectional

CSS conditions include FMS, irritable bowel syndrome (IBS), functional dyspepsia, chronic fatigue syndrome (CFS), myogenic temporomandibular disorder (TMD), tension-type headache, migraine, regional pain syndromes (myofascial pain syndrome and neck and back pain without structural pathology), restless legs syndrome, interstitial cystitis, multiple chemical sensitivity, posttraumatic stress disorder (PTSD), Gulf War syndrome, and vulvodynia [6–9]. Undoubtedly, the number will grow by the Yunus criteria for CSS [6]. Although the literature is ripe with reports of increased prevalence of various members of CSS in FMS (too many to provide references), reports on their bidirectional interrelationships are relatively few [6, 9]. Thus, while prevalence of irritable bowel syndrome (IBS) in FMS is increased compared with controls [1, 3–9], same is also true for FMS in a population of IBS and other CSS members, such as (TMD) (see below). 

Studies of the occurrence of FMS in other CSS conditions are generally limited at this time (compared with prevalence of CSS in FMS). Number of studies is indicated by the number of references (Table 1). Increased prevalence of FMS, either by the use of a healthy control group or by consideration of the prevalence of FMS in the general population, has been reported in IBS [9, 11–14], TMD [9, 15–19], headaches [9, 20–23] (including tension-type headache, migraine, and a mixed group of TTH and migraine), interstitial cystitis [9, 24–27], chronic fatigue syndrome [9, 28, 29], vulvodynia/vulvar vestiular syndrome [30, 31], and Gulf War syndrome [32–34].

Sixty-seven percent of patients who present with “idiopathic” chronic low back pain were found to have FMS [9]. Patients presenting with chronic low back or neck pain demonstrate evidence of CS [6, 8, 9], and these patients often develop widespread pain and fibromyalgia at a later time [6, 35].

MPS and FMS are overlapping syndromes [6]. There is strong evidence for CS in MPS, including decreased pain threshold by various nociceptive stimuli at sites remote from painful area, accentuated spinal nociceptive flexion reflex [6], and augmented cortical activation by functional magnetic resonance imaging (fMRI) [6, 36]. 

Most cases of FMS begin with regional pain similar to MPS. Trigger points (TrP), in addition to tender points (TP), are also present in FMS [37]. It has been suggested that a continuous input from a TrP leads to, and maintain, CS both in MPS and FMS [37]. The cause of TrP is speculative but include local trauma (including fall, motor vehicle accident, overuse, and repetitive use), spinal stress (e.g., scoliosis or poor posture), and perhaps systemic factors, for example, mental stress. TrP are likely sustained by CS [36].

## 4. FMS in Chronic Painful Diseases with Structural Pathology: An Expansion of the Fibromyalgia Territory

As early as 1983, Wolfe and Cathey recognized the concomitant occurrence of FMS among RA patients. It was an astute observation since many features of FMS, for example, pain at multiple sites (including bursal and tendon areas), fatigue, a feeling of malaise and tenderness at multiple spots, including joint areas, may also be present in RA itself. What was not definitely known at that time is whether RA patients were also tender in muscles and other sites of typical tender points. As it turned out, multiple TPs are a unique feature of FMS, although they are present to a lesser extent in other members of CSS as a manifestation of CS [6]. 

It was not until 1990s when the presence of FMS in many chronic diseases with structural pathology was generally recognized among the researchers. Now, it is known that FMS is significantly associated with RA [10, 38–41], systemic lupus (SLE) [42–48], ankylosing spondylitis (AS) [49, 50], osteoarthritis (OA) [51], diabetes mellitus [52, 53], endometriosis [54], hypothyroidism [55], and inflammatory bowel disease [56, 57] (Table 2). 

CS alone (without FM) has been reported in juvenile chronic arthritis [59], OA [60–65], endometriosis [66], carpal tunnel syndrome [67–69], chronic pancreatitis [70, 71], and Parkinson's disease [72–74]. It is likely that CS is the harbinger of future development of FMS, as may be demonstrated in future studies. It is as if “fibro, fibro everywhere and not a place for the (rational) eyes to hide.” An interesting question is, and data have not emerged yet to answer it, are other members of the CSS family, such as IBS and myogenic TMD also associated with these chronic diseases with structural pathology? 

FMS has been reported also in Sjogren's syndrome, hepatitis C, HIV, and other infections, for example, Lyme disease. True to the title of this essay, however, only those diseases that usually cause pain have been described in this paper.

## 5. Critical Evaluation of the Studies: Imperfect but the Associations Are Real

A number of studies, both in the category of FMS in CSS and FMS in DWSP, are less than satisfactory because of small numbers, unspecified or nonstandard criteria used (both for FMS and the associated diseases), an absence of, or the use of inappropriate controls as well as inappropriate statistics, for example, a failure to adjust for multiple comparisons. Lack of blindness, a critical procedure to avoid bias, was universal, as is the case with most published studies in any disease. 

However, reported prevalence rates of FMS in general were much higher than that in the general population. Since the results of all the studies converge in the same direction, that is, increased prevalence of FMS compared with study or population controls, it seems proper to conclude that true associations of FMS with the diseases discussed exist.

## 6. Pathophysiological Mechanisms of Disease Associations with FMS: Central Sensitization Is Central

### 6.1. CS and CSS Conditions

Central and common to CSS diseases are CS. The pathophysiology of CS has been discussed elsewhere [6, 8, 75] and is beyond the scope of this essay. To be sure, the mechanisms involved in CS are multiple and complex [75]. Some of these mechanisms involve temporal summation (windup), long-term potentiation (LTP), heterosynaptic potentiation, a dysfunctional descending pain inhibition, and an activation of the descending facilitatory pathway [75]. It is also evident that input in one set of nociceptive fibers amplifies subsequent response to other nonstimulated noceptive or nonnociceptive fibers (heterosynaptic potentiation). Such phenomena explain diffuse distribution of pain as well as allodynia. 

After many years of research in animal models, human volunteers, and chronic pain conditions, several facts have emerged. While a noxious stimulus at some point in the past might have initiated increased neuronal sensitivity, no further nociceptive stimulus is necessary to perpetuate and sustain a state of hyperalgesia or allodynia. That sustained pain is no longer nociceptive in nature, provoking some physicians to take recourse to such derogatory lexicons as neurotics, malingerers, somatizers, and “medically unexplained symptoms” (read “your symptoms are all in your head”). In other cases, following the adequate initial nociceptive input, only a very low level of nociceptive stimulus was needed to maintain the CS [75]. 

With regards to tender points, it is often stated that they are subjective (thus there is an issue of secondary gain here), but CS may be elicited by stimuli that do not require patient's subjective response and are thus purely objective. Such an objective “test” is nociceptive spinal flexion reflex that has been demonstrated in several CSS conditions [6]. Using ascending and random paradigms, it has been demonstrated that response bias does not play a major role in pain report during CS testing in human pain laboratory [76]. Other objective findings are brain abnormalities by neuroimaging techniques and measurements of several neurotransmitters, for example, substance P, serotonin and its metabolites, and nerve growth factor [7]. 

 There is evidence that CS is causal and not just an effect of chronicity among CSS members [6]. Asymptomatic individuals displaying CS in conjunction with genetic factors develop a CSS condition when followed up for a few years (see [6] for specific studies). Further, several centrally acting medications efficacious in FMS decrease CS [6].

The relative role of peripheral and central factors and ascending and descending pathways in fibromyalgia and other CSS diseases are not known at this time. In FMS, drugs acting on both ascending pathway (pregabalin) and descending pathway (e.g., duloxetine and milnacipran) are equally effective in general. In an editorial that evaluates several controlled studies, Martin Ingar states that descending pain control plays an important role in FMS [77]. It has been asserted that peripheral nociception is essential for CS in FMS [78]. In support of this view, the author cites a number of studies showing muscle abnormalities in FMS. Unfortunately, these studies did not include controls matched for aerobic fitness employing VO2max, nor were they blinded. Bennett and his colleagues showed that 80% of the FMS patients were not physically fit as assessed by maximal oxygen uptake (VO2max) [79]. The authors emphasized the need for using sedentary controls in FMS muscle studies.

Using appropriately matched sedentary controls using VO2max, Simms et al. showed that energy metabolism in trapezius and tibialis anterior muscles of FMS patients, including intracellular pH, was normal when compared with controls [80]. In our initial uncontrolled muscle biopsy study, mitochondrial and other changes were found [81]. However, in our subsequent activity-controlled (indirectly sedentary controlled) and blinded study, no abnormalities in the trapezius muscle were noted in FMS as compared with the controls [82] (this is the only blinded muscle biopsy study in the literature to my knowledge).

However, contribution of peripheral input (versus pathology) in FMS has been demonstrated [83]. Staud et al. showed that lidocaine injection in the trapezius muscle increased pressure pain threshold locally in both FMS patients and controls, but placebo injection did not. In addition, heat hyperalgesia of FMS patients in remote site (forearm) was also decreased, suggesting the role of peripheral input in heat hyperalgesia at a remote site (CS) [83]. However, clinical pain rating was not affected and the nature of such nociceptive locus in the muscle (e.g., inflammation and ischemia) is not clear, nor is it known if hyperalgesia from other forms of stimuli is maintained by peripheral nociception. 

In a recent placebo-controlled study [84], Affaitati et al. have shown that injection of a TrP with local anesthetic deep in the muscle tissue (the placebo group received injections near the trigger points, presumably in superficial tissue) produced a decrease in number as well as pain intensity in TrP with an increase in pain pressure threshold both in TrP and nonpainful sites. 

In the same study [84], the investigators applied hydroelectrophoresis to a FMS group having a painful joint or an area of rotator cuff partial tear using diclofenac and betamethasone in agarose gel in a tube as well as electrodes that were connected to a computerized current stimulator. The placebo group received a gel without active ingredients. The active treatment group reduced their FMS pain as well as number of TP, and the pressure pain threshold in nonpainful areas also increased. The placebo group did not demonstrate such improvement. This study also suggests that peripheral input contributes to CS. Interestingly, generalized hyperalgesia significantly improved in women with endometriosis following hysterectomy [66].

From the above discussion, it is clear that peripheral input is necessary in at least some patients. Visceral afferent input [75] may be operative in IBS [85], interstitial cystitis, and chronic pelvic pain. These patients also demonstrate somatic sensitization. Since no definite nociceptive pathology has been convincingly demonstrated in the peripheral tissue, is it possible that CS itself contributes to peripheral input in a vicious cycle? It is relatively easy to conceptualize the role of initial peripheral inflammation in cases of significant trauma incidents, for example, motor vehicle accident (MVA) and falls. In a “hit and run” phenomenon, the initial inflammation triggers CS and is maintained with little or no further peripheral input as is well accepted from experimental models [75]. 

From other experimental examples, it seems that peripheral input may not be necessary at all [75]. Such apparently “innate” CS may be due to genetics, early adverse childhood experience, prenatal stress, chemical exposure (as in multiple chemical sensitivity), dopamine deficiency (as in restless legs syndrome and Parkinson's disease), other neurotransmitter or endocrine abnormalities, past trauma as mentioned above (MVA, falls), severe and continuous sleep deprivation, and psychological trauma or stress. Other factors, for example, infection, autonomic nervous system dysfunction, inflammatory cytokines, and CNS microglia and astrocytes may contribute to CS of CSS conditions. The issue of relative role of peripheral and central factors in CS must be regarded unresolved at this time. Our best guess is that both are necessary and the relative contribution will vary from individual to individual, partly determined by genetic predisposition. 

It is interesting to speculate that there are subgroups of CS. Some may result from obvious peripheral source (such as trauma, local inflammation, neuritis, or arthritis), and others are independent of such peripheral input because of genetics, sleep deprivation, or psychological distress. Moreover, some may predominantly result from reduced descending inhibition, others from descending facilitation, and yet others from cortical or limbic activation. Future research may determine that certain drugs work predominantly in one pathway but not the others. It is entirely possible that all of the neural pathways are involved in a given individual, likely in an interacting way.

### 6.2. FMS in Chronic Painful Diseases with Structural Pathology

An obvious source of nociception is present in RA, OA, AS, SLE (associated with arthritis), endometriosis, and inflammatory bowel disease. Pain often causes poor sleep that in turn may contribute to CS. Neuritis may contribute to CS and subsequent FMS in diabetes mellitus. The mechanism of CS in hypothyroidism [55] is not obvious. However, this is a single study and needs further confirmation. RA patients with concomitant FMS have worse disease activity with joint swelling and tenderness [40, 41] and greater psychosocial distress [41]. There were no significant differences in SLE activity between SLE patients with or without FMS. FMS features, for example, fatigue and poor sleep, were more common in the SLE plus FMS group. Whether inflammation, cytokines, genetics, or endocrine factors are contributory is unknown. Concomitant presence of FMS in the above diseases has universally shown greater disability in all studies that addressed the function and overall symptom severity.

Patients with OA plus FMS had greater sleep difficulties (70%) that correlated with fatigue [51]. Those with sleep problems had more severe OA, and depression was also common. It has been suggested that repeated acute inflammation in chronic pancreatitis leads to sensitization of peripheral pancreatic nerves that subsequently lead to central neuroplasticity [86]. In Parkinson's disease, CS has been explained on the basis of somatosensory function of the basal ganglia. The role of D2 receptors in pain processing and a deficiency of dopaminergic inhibition have been suggested [72].

### 6.3. Brain Changes in Chronic Pain

Until recently, central sensitization was attributed to an abnormal function of the nociceptive/antinociceptive neurons at different levels of neuroaxis leaving the brain structure intact. The advent of magnetic resonance imaging has demonstrated that not only there is a functional reorganization of the cerebral cortex in chronic pain, for example, FMS, IBS, and chronic back pain, but also there is actual anatomic decrease in the gray matter of various regions of the brain. This imaging technique is called MR morphometry. Although areas involved vary depending on the type of chronic pain, a general picture has emerged to involve the cingulated cortex, the orbitofrontal cortex, the insula, and the dorsal pons, representing a common “brain signature” [87]. The anterior cingulate cortex (ACC) plays a particularly important role in pain modulation and analgesia. ACC interacts with orbitofrontal cortex, PAG, and the amygdala. Together, it plays a crucial role in endogenous pain control. An anatomic shrinkage thus may contribute to enhanced sensitivity in chronic pain. 

In FMS, a reduction in gray matter has been demonstrated in left parahippocampal gyrus, cingulated gyrus, insula, and medial frontal cortex. A decrease in gray matter involves simple reduction of cell size or atrophy of the neurons or the glia and does not necessarily imply neuronal destruction. Thus, with proper treatment of chronic pain, the gray matter may regain its original size [87]. An important question is whether continued and prolonged chronic pain will lead to irreversible degeneration. A crucial issue is whether the morphometric changes with atrophy of the gray matter are the cause or consequence of chronic pain. It seems that the changes result from ongoing CS. If so, centrally acting medications that diminish CS may retard or even reverse the gray matter change.

## 7. Significance of Disease Associations with FMS: Implication for Science and Patient Care

The concept of mutual association of the CSS has helped to better diagnose these conditions without extensive and unnecessary investigations. Recognition of an objective pathophysiological basis has contributed to a better understanding and treatment as well as physician acceptance of these common problems that cause much distress to our patients. Thus, it has helped us to understand why nonsteroidal anti-inflammatory drugs that act peripherally are not efficacious in FMS and why centrally acting medications as well as nonpharmacologic approach, for example, cognitive behavioral therapy (that act centrally), should be the appropriate management approach. Since total disease burden with functional impairment is greater in those with many associated conditions, a practicing physician should treat all these conditions for optimal results. 

Since pathophysiologically the CSS disorders are similar, discovery of a certain mechanism and effective medication in one disease may be applied to others. In developing new therapy, the effect of a new medication on CS may be investigated before undertaking expensive clinical trials. 

The most important implication of concomitant FMS in chronic diseases with structural pathology is its recognition for optimal management. For example, when FMS symptoms (e.g., increased pain and fatigue) with multiple tender points are present in RA or SLE, they should not be automatically attributed to increased activity of these diseases and one should not prescribe higher doses of a biologic agent or corticosteroids without proper TP examination and laboratory evaluation. Appropriate attention should be directed to the management of FMS with centrally acting medications, cognitive behavioral therapy, and management of sleep problems. A patient with severe pain in OA will require both peripherally acting analgesics as well as those that act centrally, such as cyclobenzaprine, pregabalin, duloxetine, and milnacipran.

## 8. Summary: A Few Words to Store and Ponder

CSS diseases are based on a neurochemical pathology, and they are not psychological or psychiatric illnesses. Associated psychosocial issues, as may be present in any chronic disease, including RA or SLE or cancer should be addressed. CSS conditions are mutually associated, and a particular patient may have several of them. Multiple symptoms in these patients have a demonstrable pathophysiological basis and do not represent somatization. It is most important that FMS is suspected in all chronic diseases with structural pathology, for example, RA, SLE, OA, and AS, so that proper diagnosis and management can be undertaken. 

## Figures and Tables

**Table 1:** Prevalence of fibromyalgia syndrome (FMS) in other central sensitivity syndromes (CSS) conditions.

CSS condition*	% prevalence of FMS (mean)	% prevalence of FMS (range)	Reference no.
Irritable bowel syndrome	40.7	20.0–65.0	[9, 11–14]
Temporomandibular disorder	23.7	13.0–52.0	[9, 15–19]
Headaches (all)	26.3	10.0–40.0	[9, 20–23]
Tension-type headache	29.7	23.0–36.4	[9, 23]
Migraine	16.0	10.0–22.0	[21, 22]
Mixed^¶^	38.2	36.4–40.0	[20, 23]
Interstitial cystitis	15.4	12.0–22.4	[9, 24–27]
Chronic fatigue syndrome	55.2	15.6–80	[9, 28, 29]
Vulvar vestibular syndrome	23.4	15.6–31.2	[30, 31]
Gulf War syndrome	17.6	2.0–33.8	[32–34]

*Prevalence of FMS in other CSS conditions with a single study has been discussed in the text.

^¶^Mixture of tension-type headache and migraine.

**Table 2:** Prevalence of fibromyalgia syndrome (FMS) in chronic painful diseases with structural (organic) pathology (DWSP).

Chronic DWSP	% prevalence of FMS (mean)	% prevalence of FMS (range)	Reference no.
Rheumatoid arthritis	15.4	12.2–19.8	[10, 38–41]
SLE	16.2	5.0–25.3	[42–48, 58]
Ankylosing spondylitis*	30.4	10.8–50.0	[49, 50]
Osteoarthritis	11.0	—	[51]
Diabetes mellitus*	17.5	17.0–18.0	[52, 53]
Endometriosis	5.9	—	[54]
Hypothyroidism	34.0	—	[55]
Crohn's disease	26.0	3.0–49.0	[56, 57]
Ulcerative colitis	11.4	3.7–49.0	[56, 57]

SLE: systemic lupus erythematosus.

*Female patients only.
